# Systematic review of cost effectiveness and budget impact of artificial intelligence in healthcare

**DOI:** 10.1038/s41746-025-01722-y

**Published:** 2025-08-26

**Authors:** Rabie Adel El Arab, Omayma Abdulaziz Al Moosa

**Affiliations:** Almoosa College of Health Sciences, Alhsa, Saudi Arabia

**Keywords:** Health care, Health care economics

## Abstract

This systematic review examines the cost-effectiveness, utility, and budget impact of clinical artificial intelligence (AI) interventions across diverse healthcare settings. Nineteen studies spanning oncology, cardiology, ophthalmology, and infectious diseases demonstrate that AI improves diagnostic accuracy, enhances quality-adjusted life years, and reduces costs—largely by minimizing unnecessary procedures and optimizing resource use. Several interventions achieved incremental cost-effectiveness ratios well below accepted thresholds. However, many evaluations relied on static models that may overestimate benefits by not capturing the adaptive learning of AI systems over time. Additionally, indirect costs, infrastructure investments, and equity considerations were often underreported, suggesting that reported economic benefits may be overstated. Dynamic modeling indicates sustained long-term value, but further research is needed to incorporate comprehensive cost components and subgroup analyses. These findings underscore the clinical promise and economic complexity of AI in healthcare, emphasizing the need for context-specific, methodologically robust evaluations to guide future policy and practice effectively.

## Introduction

Artificial Intelligence (AI) refers to a suite of computational techniques that enable machines to perform tasks typically requiring human intelligence^1^. These techniques include, but are not limited to, machine learning and deep learning—approaches that have been widely applied in healthcare for predictive analytics, diagnostic support, and personalized treatment planning. In addition, generative AI, which involves models capable of producing novel content based on learned data, is emerging as a promising tool in various clinical applications^2–4^.

The rapid evolution of AI in medicine is transforming the landscape of healthcare delivery. In recent years, advances in machine learning and deep learning have led to the development of sophisticated AI applications capable of enhancing diagnostic precision, personalizing treatment strategies, and streamlining clinical workflows^5^. As these technologies transition from experimental prototypes to integral components—defined here as technologies routinely implemented and widely adopted within clinical practice, often accompanied by formal regulatory approval—they promise to revolutionize patient care across diverse specialties^6^. However, while the clinical benefits associated with AI in healthcare are widely acknowledged, the economic implications remain less clearly articulated. Given the substantial investment required for the integration of AI into clinical practice, assessing its economic viability becomes crucial. Although improvements in clinical outcomes attributable to AI have been extensively documented, a rigorous evaluation of the economic dimensions, including affordability, sustainability, and cost-effectiveness—is essential to support informed decision-making among healthcare leaders and policymakers. Against the backdrop of escalating healthcare costs driven by aging populations, chronic disease burdens, and technological innovation, it is imperative to assess whether the financial investments in AI yield sustainable benefits and represent good value for money^7–10^.

AI’s integration into clinical practice spans multiple disciplines, including radiology, oncology, cardiology, and primary care. For instance, deep learning algorithms applied to medical imaging have demonstrated diagnostic accuracies that rival—and in some cases surpass—that of expert clinicians^11^. In oncology, AI-based prognostic models have been used to stratify patient risk more accurately, enabling earlier interventions and more tailored treatment regimens^12^. These advancements underscore the potential for AI to fundamentally improve clinical outcomes and reduce the burden on healthcare providers. Yet, the implementation of such technologies requires not only a rigorous demonstration of clinical efficacy but also a clear understanding of their economic impact. Nonetheless, moving beyond clinical effectiveness alone, stakeholders including healthcare policymakers, administrators, and clinicians must clearly understand whether these promising AI applications are economically justifiable.

Globally, healthcare expenditure is rising at an unsustainable rate. Factors such as demographic shifts, the increasing prevalence of chronic diseases, and the complexity of modern medical care have all contributed to growing financial pressures on health systems^13–15^. In this context, the potential for AI to streamline processes and reduce costs has garnered significant interest. Economic evaluations encompass distinct analytical approaches, primarily categorized into full economic evaluations—including cost-effectiveness analysis (CEA), cost-utility analysis (CUA), and cost-benefit analysis (CBA) and partial evaluations, such as budget impact analysis (BIA). Full economic evaluations systematically compare both costs and outcomes (e.g., clinical effectiveness or quality-adjusted life years) of two or more interventions, aiming to determine value for money or optimal resource allocation. In contrast, budget impact analysis evaluates solely the financial consequences of adopting a new intervention within a specific healthcare setting or payer budget, without explicitly measuring clinical effectiveness or broader societal outcomes^16,17^. Moreover, identifying key economic determinants—such as technology acquisition costs, implementation expenses, and workflow integration challenges—can help tailor AI deployments to local contexts. Two systematic reviews have previously explored economic evaluations of AI interventions in healthcare. Voets et al.^18^ systematically assessed economic evaluations published between 2016 and 2021, primarily focusing on AI applications related to automated medical imaging analysis, revealing methodological inconsistencies and limited rigor in long-term economic modeling approaches. Vithlani et al.^19^ subsequently reviewed a broader range of AI interventions, highlighting persistent gaps, including reliance on traditional static models, insufficient transparency in reporting AI-specific costs, and inadequate consideration of evolving AI capabilities over time.

However, these reviews did not explicitly synthesize or evaluate the economic impact of AI interventions within specific clinical contexts, nor did they systematically address how variations across healthcare settings affect economic outcomes. The present systematic review expands upon these earlier efforts by explicitly focusing on clinical AI interventions integrated across diverse healthcare settings—including hospitals, primary care, and community clinics—and by conducting an in-depth thematic synthesis that directly connects economic evidence to clinical performance, implementation challenges, and contextual factors influencing economic sustainability and equity. This review aims to systematically evaluate the economic outcomes—including cost-effectiveness, utility, and budget impact—of clinical AI interventions across diverse healthcare settings and domains, with particular emphasis on hospitals, primary care, and community-based systems. These evaluations span a wide range of clinical specialties, including oncology, cardiology, ophthalmology, dentistry, infectious diseases, intensive care management, and diagnostic imaging. In doing so, the review examines how AI applications compare to traditional diagnostic or management approaches in terms of both clinical effectiveness and associated cost metrics. It also explores the key factors that influence cost-effectiveness, such as technology acquisition, implementation and maintenance costs, workflow integration, and variations in healthcare system financing. By synthesizing these dimensions, the review provides a comprehensive understanding of the economic value and practical feasibility of AI in clinical care.

## Results

**Fig. 1:**
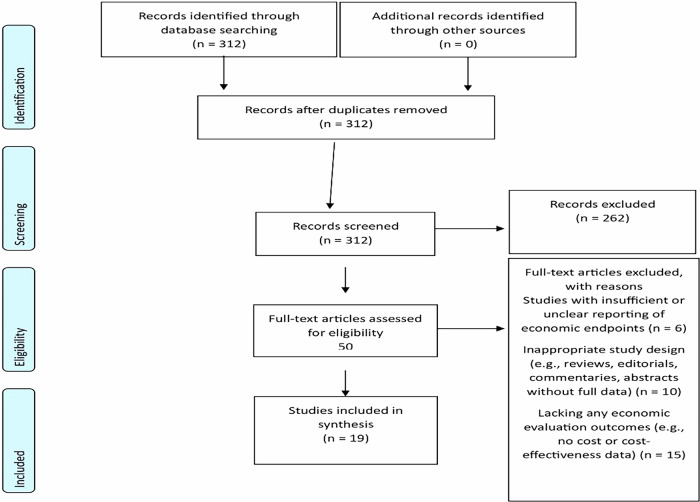
PRISMA 2020 flow diagram. This diagram illustrates the systematic review process, detailing each stage—from record identification through screening, eligibility assessment, and final inclusion—with explicit reasons for exclusion at various stages in accordance with the PRISMA 2020 guidelines.

Our systematic screening began with 312 unique records retrieved from PubMed, Embase, Web of Science and the Cochrane Library. After duplicate removal, all 312 records underwent title and abstract screening, resulting in 262 exclusions. Fifty full-text articles were assessed for eligibility; 31 were excluded (6 for unclear or insufficient economic reporting, 10 for ineligible study design, and 15 for lacking any economic outcomes), yielding 19 studies for inclusion (see Fig. 1).

Our synthesis encompasses 19 studies published between 2020 and 2024^20–38^, across diverse clinical domains—including oncology, atrial fibrillation screening, gastrointestinal endoscopy, diabetic retinopathy screening, dental caries detection, ICU management (discharge and sepsis detection), medication management, tuberculosis screening and treatment monitoring, glaucoma screening, lung cancer screening, and breast cancer screening—and conducted in North America, Europe, and Asia. The studies applied a variety of economic evaluation methods: cost-effectiveness analysis (CEA; *n* = 14), cost-utility analysis (CUA; *n* = 4), cost-minimization analysis (CMA; *n* = 2), and budget impact analysis (BIA; *n* = 1). Analytical perspectives also varied, with 13 studies adopting a healthcare system perspective, 4 a societal perspective, and 1 a payer perspective. The evaluations ranged from short-term follow-ups (90 days) to lifetime projections, all utilizing discount practices in line with regional guidelines.

Direct medical costs, including diagnostic, procedural, and treatment expenses, were consistently reported across all studies, while indirect costs were less frequently included (*n* = 4). Although most studies employed standard methods for currency conversion and establishing cost-year baselines, some did exhibit incomplete reporting in these areas. The differentiation of study objectives, outcomes, and time horizons provided nuanced insights into AI’s economic viability across various contexts (see Table 1: Summary of Included Studies). Additionally, Supplementary Table 1 offers a comprehensive classification of each study by modeling type, intervention area, and dynamic status.

**Table 1:** Summary of included studies

Study (Author et al. (year))	Study characteristic (country; setting; study design)	Population & AI intervention (target; AI system; comparator; clinical context)	Economic evaluation methodology (type; perspective; time horizon; discounting)	Key outcomes & findings
Areia et al. ^33^	• Country: Multinational • Setting: Endoscopy units performing screening colonoscopy • Study Design: Modeling study using decision-analytic/Markov methods	• Target: Average-risk individuals undergoing colonoscopy • AI System: AI-aided polyp detection and optical diagnosis • Comparator: Standard colonoscopy (no AI) • Context: Optimize detection & reduce unnecessary procedures	• Type: Cost-effectiveness analysis (CEA) • Perspective: Healthcare system/payer • Time Horizon: Modeled over screening interval/lifetime • Discounting: Standard (e.g., 3–5% per annum, as assumed)	• AI integration shown to be cost-effective and potentially cost-saving by improving diagnostic accuracy and efficiency
de Vos et al. ^32^	• Country: Netherlands • Setting: Intensive Care Units (ICUs) • Study Design: Economic modeling study using decision analytic/Markov models	• Target: ICU patients eligible for discharge • AI System: ML tool to predict and prevent untimely ICU discharge • Comparator: Standard intensivist-led discharge decisions • Context: Optimize discharge timing and reduce readmissions	• Type: Cost-effectiveness analysis (CEA/CUA) • Perspective: Healthcare system (possibly societal) • Time Horizon: Short-to-medium term (e.g., hospitalization/1 year) • Discounting: As per local guidelines (e.g., 3–4%)	• ML tool demonstrated potential cost savings by preventing premature discharge and reducing readmissions
Ericson et al. 2022	• Country: Sweden • Setting: ICUs across Sweden • Study Design: Decision analysis/modeling study (decision tree–based economic model)	• Target: ICU patients at risk for sepsis • AI System: ML algorithm for early sepsis detection • Comparator: Standard clinical practice (physician assessment + labs) • Context: Early sepsis detection to reduce ICU LOS and mortality	• Type: Cost-effectiveness analysis (CEA) • Perspective: Swedish healthcare system • Time Horizon: 1-year model • Discounting: ~3% per annum	• AI intervention estimated to save ~€76 per patient and yield substantial national savings by reducing ICU days and improving outcomes
Gomez Rossi et al. ^29^	• Country: Multinational (US for dermatology; Germany for dentistry; Brazil for ophthalmology) • Setting: Dermatology clinics, dental clinics, ophthalmology screening programs • Study Design: Markov model–based economic evaluation with Monte Carlo microsimulations	• Target: – Dermatology: US adults (~50 years) for melanoma – Dentistry: German children (age 12) for caries detection – Ophthalmology: Brazilian diabetic patients (~40 years) for DR grading • AI System: Decision-support systems aiding detection/grading • Comparator: Standard clinical detection • Context: Enhance diagnostic accuracy and reduce misdiagnoses	• Type: Cost-effectiveness analysis (CEA) • Perspective: Country-specific (US: mixed payer; Germany & Brazil: public) • Time Horizon: Lifetime • Discounting: 3% per annum	• AI was cost-saving in melanoma (ICER = –$27,580/QALY) and dentistry (ICER = –€15 per tooth retention year); in diabetic retinopathy, AI was more expensive with similar QALYs
Hendrix et al. ^21^	• Country: Global • Setting: Multiple clinical settings (hospitals, outpatient, community) • Study Design: Conceptual/theoretical analysis and framework development	• Target: Broad – healthcare systems, clinicians, and patients impacted by AI • AI System: Various clinical AI applications (diagnostic, decision support, workflow automation) • Comparator: Standard human-driven care • Context: Evaluate overall economic value of clinical AI	• Type: Conceptual framework incorporating CEA, CUA, CBA, CMA • Perspective: Multi-level (healthcare system, societal, payer, patient) • Time Horizon: Varies by application • Discounting: Not explicitly detailed	• AI’s value depends on integration, generalizability, and productivity improvements; challenges (regulatory, equity) and opportunities are identified
Hill et al. ^26^	• Country: United Kingdom • Setting: Primary care within the NHS • Study Design: Economic modeling study combining decision tree and Markov modeling	• Target: Adults aged ≥50 years at risk for undiagnosed AF • AI System: ML risk prediction algorithm using clinical and demographic data • Comparator: Systematic (age-based) and opportunistic screening • Context: Early AF detection for stroke prevention	• Type: Cost-effectiveness analysis (CEA/CUA) • Perspective: NHS (healthcare system) • Time Horizon: Lifetime • Discounting: ~ 3.5% per annum	• ML-targeted screening produced ICERs between £4847 and £5544 per QALY and required fewer tests compared to standard methods
Huang et al. ^36^	• Country: China (rural areas) • Setting: Community health stations and rural hospitals • Study Design: Modeling study using decision tree and Markov models	• Target: Diabetic patients in rural areas • AI System: Deep learning–based DR screening tool • Comparator: Ophthalmologist screening and no screening • Context: Improve early DR detection where specialists are scarce	• Type: Cost-effectiveness analysis (CEA) using QALYs • Perspective: Healthcare system and societal • Time Horizon: 35 years • Discounting: 3% per annum	• Achieved an ICER of ~$1,108 per QALY and dominated ophthalmologist screening, thereby reducing long-term costs
Karabeg et al. ^24^	• Country: Norway • Setting: Clinical screening center during Minority Women’s Day • Study Design: Pilot cross-sectional cost-analysis study	• Target: 33 minority women with diabetes (66 eyes) • AI System: EyeArt® automated DR detection system • Comparator: Manual DR grading by ophthalmologists • Context: Improve DR screening in an underrepresented high-risk group	• Type: Cost-minimization analysis (CMA) • Perspective: Healthcare system and payer • Time Horizon: Single screening episode • Discounting: Not applicable	• Reduced cost per patient by ~52% (from ~$273 to ~$131) with near-perfect diagnostic agreement
Kessler et al. ^27^	• Country: United States (California, Medicaid) • Setting: Health plan-level medication management • Study Design: Retrospective observational study using regression methods	• Target: 2150 high-risk Medicaid members (aged 40–64 with multiple chronic conditions) • AI System: Surveyor Health AI platform for medication risk assessment and management • Comparator: Standard medication management • Context: Improve adherence and reduce hospital/ED utilization	• Type: Cost-effectiveness analysis with ROI calculation • Perspective: Healthcare payer (Medicaid) • Time Horizon: 21 months • Discounting: Not applied	• Reported savings of $14 million annually) and an ROI of 12.4:1
Mital & Nguyen^34^	• Country: United States • Setting: Population-based breast cancer screening simulation • Study Design: Model-based economic evaluation using decision tree and microsimulation	• Target: Hypothetical cohort of white women aged 40–49 • AI System: Deep learning–based risk prediction for screening stratification • Comparator: Polygenic risk score (PRS) and family history-based stratification • Context: Optimize screening frequency and reduce over-screening	• Type: Cost-effectiveness analysis (CEA) • Perspective: Healthcare system • Time Horizon: Lifetime • Discounting: 3% per annum	• Achieved an ICER of ~$23,755 per QALY, rendering AI-guided screening cost-effective compared to universal annual screening
Mori et al. ^20^	• Country: Multinational • Setting: Colonoscopy screening in endoscopy units • Study Design: Retrospective add-on analysis of a clinical trial	• Target: Patients undergoing colonoscopy for screening/surveillance • AI System: AI-aided polyp diagnosis software • Comparator: “Resect-all” strategy (removal of all polyps for histopathology) • Context: Enhance efficiency and reduce unnecessary procedures	• Type: Combined cost-minimization and cost-effectiveness analysis • Perspective: Healthcare system and payer • Time Horizon: Per-procedure analysis with annual projections • Discounting: Not applied	• Reduced procedural costs (e.g., an 18.9% reduction in Japan) and showed potential for substantial national savings
Murtojärvi et al. ^22^	• Country: Finland • Setting: Clinical oncology centers and hospital registries • Study Design: Comparative modeling study using penalized Cox regression (Greedy and LASSO algorithms)	• Target: Patients with metastatic castration-resistant prostate cancer • AI System: Prognostic models using Greedy and LASSO feature selection • Comparator: Full-variable Cox regression model • Context: Enhance survival prediction while reducing diagnostic costs	• Type: Cost-effectiveness analysis (CEA) • Perspective: Healthcare system • Time Horizon: Lifetime • Discounting: Not explicitly applied	• Greedy algorithm was more cost-effective at lower budgets; LASSO performed better at higher budgets without sacrificing accuracy
Nsengiyumva et al. ^31^	• Country: Pakistan • Setting: TB clinic in Karachi (Indus Health Network) • Study Design: Decision analysis model	• Target: Adults ≥15 years with TB symptoms (HIV-negative) • AI System: Deep learning–based CXR interpretation • Comparator: Standard microbiological testing without AI triage • Context: Prioritize high-risk patients for confirmatory testing	• Type: Cost-effectiveness analysis (CEA) • Perspective: Healthcare provider • Time Horizon: 1 year • Discounting: Not applied	• Achieved ICERs of ~$39–$40 per DALY averted; reduced diagnostic costs by 19–37% per 1000 persons screened
Salcedo et al. ^30^	• Country: United States (Los Angeles County) • Setting: TB treatment monitoring within a public health program • Study Design: Markov model-based cost-effectiveness analysis	• Target: Adults with active TB (non-MDR, HIV-negative) • AI System: AiCure for remote treatment adherence monitoring • Comparator: Traditional directly observed therapy (DOT) (and VDOT scenario) • Context: Improve adherence and reduce resource use	• Type: Cost-effectiveness analysis (CEA) • Perspective: US healthcare system • Time Horizon: 16 months • Discounting: Not applied	• AiCure was dominant, saving approximately $2226 per patient with slightly higher QALYs (1.05 vs. 1.03)
Schwendicke et al. ^38^	• Country: Germany • Setting: Dental clinics and radiology centers using bitewing radiographs • Study Design: Markov model-based economic evaluation	• Target: Individuals aged ≥12 years needing proximal caries detection • AI System: U-Net deep learning model • Comparator: Traditional dentist-based radiographic assessment • Context: Early detection to improve tooth retention and reduce invasive treatments	• Type: Cost-effectiveness analysis (CEA) using tooth retention years • Perspective: German healthcare system • Time Horizon: Lifetime • Discounting: 3% per annum	• Improved tooth retention (64 vs. 62 years) and reduced per-patient costs (e.g., €298 vs. €322); negative ICER indicates cost savings
Szymanski et al. ^28^	• Country: United Kingdom • Setting: Primary care practices within the NHS • Study Design: Budget Impact Analysis (BIA) using a national population model	• Target: Adults aged ≥65 years without known AF • AI System: ML algorithm for predicting high AF risk using routine clinical data • Comparator: Standard opportunistic screening (pulse palpation + ECG) • Context: Early AF detection to prevent strokes	• Type: Budget Impact Analysis (BIA) • Perspective: UK NHS and Personal Social Services (PSS) • Time Horizon: 3 years • Discounting: Not applied	• ML-only screening projected to save ~£80.4 million over 3 years; combined strategies further improve cost savings
van Leeuwen et al. ^37^	• Country: United Kingdom • Setting: Acute stroke care in the NHS (using CTA imaging) • Study Design: Early Health Technology Assessment using a Markov model	• Target: Patients with suspected ischemic stroke undergoing CTA • AI System: AI-assisted detection of large vessel occlusions (LVOs) • Comparator: Standard radiologist interpretation • Context: Improve stroke diagnosis and reduce treatment delays	• Type: Cost-effectiveness/Cost-utility analysis (CEA/CUA) • Perspective: Societal (UK NHS & PSS) • Time Horizon: Lifetime • Discounting: 4% for costs; 1.5% for QALYs	• AI intervention yielded net savings of ~$156 per patient and projected NHS-wide savings of ~$11 million annually; considered dominant
Xiao et al. ^35^	• Country: China (Changjiang County, Hainan Province) • Setting: Community-based screening in a rural public health system • Study Design: Markov model-based cost-offset analysis	• Target: Elderly individuals aged ≥65 years in remote areas • AI System: Automated glaucoma screening from fundus images with ophthalmologist confirmation • Comparator: No screening (standard opportunistic detection) • Context: Early detection to prevent progression to PACG and blindness	• Type: Cost-offset analysis • Perspective: Chinese healthcare system • Time Horizon: 15 years • Discounting: 5% per annum	• Cumulative incremental cost over 15 years was ~$434,903; incremental cost ~$1,464 per PACG case prevented; blindness reduced by ~33.3%
Ziegelmayer et al. ^25^	• Country: Germany • Setting: Hospital-based lung cancer screening using low-dose CT (LDCT) • Study Design: Markov model-based CEA with probabilistic sensitivity analysis	• Target: High-risk individuals (USPSTF criteria: age 55–80, ≥20 pack-years) • AI System: Deep-learning–assisted nodule detection and classification on LDCT scans • Comparator: Standard LDCT screening (without AI) • Context: Improve nodule detection and reduce false positives	• Type: Cost-effectiveness analysis (CEA) • Perspective: Healthcare provider (German system) • Time Horizon: 20 years • Discounting: 3% per annum	• AI-assisted LDCT produced an average saving of ~$68 per patient and remained cost-saving (dominant) up to an AI cost threshold of ~$1240 per scan

Although dynamic modeling (incorporating learning curves and time-dependent improvements) is essential to accurately reflect the evolving performance of AI systems, it is important to note that approximately 63% (12\19) of the studies in our review are based on dynamic or semi-dynamic models (as detailed in Table 1 and Supplementary Table 1). In contrast, the remaining studies rely on static decision-analytic models (7\19), which may lead to overestimated outcomes due to fixed transition probabilities and the absence of adaptive learning effects. This differentiation is crucial because it suggests that while many evaluations already incorporate adaptive features, the cost-effectiveness estimates from static models might present an overly optimistic picture of AI’s economic benefits.

The thematic synthesis reveals three interrelated themes that collectively encapsulate the multifaceted economic impact of clinical AI interventions. First, the Comparative Economic Outcomes theme. Second, the Key Cost Drivers and Sensitivity Analyses theme. Third, the Economic Impact and Implementation Considerations theme Together, these themes provide a comprehensive framework for understanding the economic advantages and challenges of integrating AI into clinical practice. The thematic synthesis directly reflects the research aim by identifying and analyzing the key components influencing the cost-effectiveness of clinical AI interventions, across diverse clinical domains and economic evaluation types. This includes both quantitative outcome measures (e.g., ICERs, QALYs, cost savings) and qualitative determinants such as implementation barriers, workflow integration, and regional cost variability.

### Comparative economic outcomes

Several studies demonstrate that AI interventions not only achieve comparable clinical outcomes to standard care but also generate measurable improvements in QALYs and other effectiveness metrics. QALY estimates were most frequently derived from published utility weights aligned with patient-reported outcome measures such as EQ-5D or SF-6D, which reflect health-related quality of life over specified time horizons. For example, Hill et al.^26^ and van Leeuwen et al.^37^ used lifetime QALY projections based on stroke or atrial fibrillation risk reduction. In addition to QALYs, other effectiveness metrics reported across studies included DALYs averted^31^, diagnostic yield improvements^38^, clinical event prevention^34^, and years of life gained^22^, offering a multidimensional view of the clinical impact of AI interventions.”

Murtojärvi et al.^22^, for instance, demonstrated significant cost reductions in oncology through AI-driven feature selection, aligning closely with the overarching theme of improved economic performance through enhanced clinical precision and resource utilization. Similarly, in atrial fibrillation (AF) screening, Hill et al.^26^ reported that an ML-based risk prediction algorithm achieved ICERs ranging between £4847 and £5544 per QALY gained—substantially lower than the NHS threshold of £20,000 per QALY gained—by effectively reducing the number of screenings required^26^.

In diabetic retinopathy screening, both Xie et al.^23^ and Huang et al.^36^ found that AI-driven models reduced per-patient screening costs by 14–19.5% and achieved ICERs as low as $1107.63 per QALY. These improvements were observed in settings as diverse as Singapore’s national programme and rural China, demonstrating AI’s robust clinical impact across resource-rich and resource-limited environments^23,36^. Additionally, Schwendicke et al.^38^ reported that AI-assisted dental caries detection improved tooth retention (64 years vs. 62 years) and achieved an ICER of –€13.9 per additional year of tooth retention, underscoring that AI can enhance clinical outcomes while reducing long-term treatment costs^38^.

Cost-savings at both the patient and system levels are consistently reported. Mori et al.^20^ reported annual national savings of $149.2 million in Japan and $85.2 million in the United States from an AI-assisted colonoscopy strategy, without a formal ICER but indicating dominance- consistent with the identified theme of AI-driven economic efficiency. In medication management, Kessler et al.^27^ documented a return on investment (ROI) of 12.4:1, representing economic efficiency rather than cost-effectiveness^27^. In breast cancer screening, Mital and Nguyen estimated an ICER of $23,755 per QALY gained for an AI-guided breast cancer screening strategy, well below the $100,000/QALY benchmark^34^. Szymanski et al.^28^ further estimated that ML-based AF screening in the UK could reduce NHS and Personal Social Services (PSS) costs by up to –£80.4 million over three years^28^. Salcedo et al.^30^ demonstrated AI dominance in TB monitoring, with cost savings of $2226 per patient and slightly higher QALYs (1.05 vs. 1.03).

#### Key cost drivers and sensitivity analyses

This theme specifically interrogates the cost components driving economic performance, aligning with our objective to evaluate the elements most critical to AI’s cost-effectiveness. Across the reviewed studies, we identified four principal categories of cost components that significantly influenced cost-effectiveness outcomes: (1) technology acquisition costs, (2) implementation and integration costs, (3) ongoing maintenance and support, and (4) indirect costs such as productivity losses or avoided downstream complications.

The unit cost of AI technology and its implementation plays a decisive role in overall economic outcomes.

Mori et al.^20^ emphasized that maintaining AI implementation costs at approximately $19 per colonoscopy procedure is crucial for achieving cost-savings. In lung cancer screening, Ziegelmayer et al.^25^ reported that AI-assisted low-dose computed tomography (LDCT) remained cost-saving (saving $68 per patient) if the cost per scan stayed below $1240—a threshold confirmed through extensive probabilistic sensitivity analyses^25^. Hendrix et al.^21^ emphasized the critical need for controlling operational expenses, directly underpinning the thematic focus on cost drivers influencing AI sustainability.

Diagnostic accuracy—measured by sensitivity, specificity, and AUROC—is a recurrent determinant of cost-effectiveness. For instance, Hill et al.^26^ illustrated how incremental improvements in diagnostic precision markedly reduced unnecessary interventions and improved economic outcomes, exemplifying the core analytical insight regarding the significance of clinical accuracy within economic analyses. In dental caries detection, Schwendicke et al.^38^ demonstrated that higher sensitivity (0.75 vs. 0.36 for traditional assessments) led to earlier interventions and a corresponding reduction in long-term treatment costs^38^. In DR screening, the performance differences between semi-automated and fully automated AI models—as shown by Xie et al.^23^ and Huang et al.^36^ —directly impacted cost per patient and ICERs, emphasizing that even small improvements in diagnostic accuracy can have significant economic implications.

Efficient integration of AI into existing clinical workflows and local variations in healthcare financing further influence economic outcomes. Kessler et al.^27^ illustrated that streamlined AI-assisted medication management led to reduced hospitalizations and overall cost savings by optimizing pharmacist workflow. Similarly, regional cost differences are highlighted in studies on DR screening, where Singapore^23^ and rural China^35^ reported how optimized integration and regional economic context directly influenced the generalizability of AI benefits, reinforcing the thematic understanding of contextual variability in AI economic evaluations. In breast cancer screening, Mital and Nguyen^34^ underscored that even minor variations in AI cost per mammogram (with a threshold of $318) could determine whether AI remains cost-effective compared to polygenic risk scores. Nearly every study conducted extensive sensitivity analyses to test the robustness of their economic evaluations. Ziegelmayer et al.^25^ used up to 30,000 Monte Carlo simulations to demonstrate that AI-assisted LDCT remained cost-saving under various scenarios. In the TB domain, Nsengiyumva et al.^31^ confirmed that AI-based CXR triage remained dominant when diagnostic specificity was maintained above 80%, while Salcedo et al.^30^ found that AiCure was cost-saving in 96.4% of simulations at a $150,000/QALY threshold. Likewise, sensitivity analyses in the AF screening study (Hill et al.^26^) and the breast cancer screening evaluation (Mital and Nguyen)^34^ confirmed that the economic benefits of AI are robust as long as key parameters (e.g., diagnostic accuracy, technology costs) remain within specified ranges^25,26,30,31,34^.

These components collectively shaped the economic value of AI interventions and were consistently highlighted in studies that reported favorable cost-effectiveness, underscoring their central role in determining AI’s viability and alignment with our study’s objectives

#### Economic impact and implementation considerations

Overall, the synthesis indicates that clinical AI interventions are highly cost-effective or even cost-saving across many applications. For example, studies in AF screening^26^, colonoscopy^20^, and medication management^27^ consistently reported low ICERs and substantial per-patient cost reductions relative to conventional care. However, not all applications yield net savings: for instance, while AI in glaucoma screening improved clinical outcomes by reducing blindness by 33.3%, it resulted in additional long-term costs^35^. Similarly, a multi-specialty evaluation by Gomez Rossi et al.^29^ demonstrated that although AI was dominant in melanoma and dental caries detection, its economic value in diabetic retinopathy screening in Brazil was less favorable—indicating that, in this context, the intervention was cost-effective (meeting acceptable ICER thresholds) but not cost-saving. These nuances underscore that the economic benefits of AI are highly context-dependent and must be interpreted according to the specific healthcare settings, cost structures, and outcome measures involved.

The cumulative evidence supports the broader adoption of clinical AI interventions, particularly in domains where low technology costs, high diagnostic accuracy, and streamlined workflows converge to deliver both clinical and economic benefits^21,22,26^. Regional differences in cost structures—as observed in studies from Singapore^23^, rural China^35^, and the UK^28^—suggest that local adaptations and context-specific evaluations are necessary to guide reimbursement and implementation decisions effectively. Moreover, while contextual adaptation is critical to ensuring economic feasibility, it also has direct implications for health equity. Regional cost variations and disparities in digital infrastructure can either enable or hinder equitable access to AI technologies. Karabeg et al.^24^ conducted in underserved minority populations, emphasizes the importance of designing and evaluating AI interventions that are inclusive, demographically representative, and accessible across all care settings.

We assessed the reporting quality of the 19 included studies using the CHEERS 2022 checklist, which consists of 28 items covering key domains such as study objectives, target population, setting, study perspective, comparators, time horizon, discounting, outcome measurement, resource use, cost estimation, model assumptions, analytic methods (including sensitivity analyses), and disclosures. Overall, the studies demonstrated high reporting quality, with total scores ranging from 21/28 to 27/28. A detailed summary of the CHEERS 2022 scores for each study is provided in Supplementary Table 2.

We further used the Drummond checklist, which comprises 10 critical items. These items assess whether each study clearly defined its research question and alternatives, established clinical effectiveness, comprehensively identified, measured, and valued costs and outcomes, performed appropriate incremental analyses (e.g., ICER calculation), and thoroughly addressed uncertainty through sensitivity analyses. Overall, the studies scored between 8/10 and 9/10. A detailed breakdown of the Drummond checklist scores for each study is presented in Supplementary Table 3.

Taken together, the CHEERS 2022 and Drummond assessments indicate that the overall quality and methodological rigor of the 19 included economic evaluations are high. Most studies reported a substantial majority of the CHEERS items (between 21 and 27 out of 28), and nearly all met 8–9 of the 10 Drummond criteria. The strengths in both reporting transparency and methodological execution enhance our confidence in the synthesized economic evidence. Minor gaps—in areas such as indirect cost valuation or specific cost conversion details—were noted but did not undermine the overall robustness of the findings.

## Discussion

This systematic review synthesized economic evidence from 19 diverse clinical AI interventions across multiple specialties, demonstrating consistent clinical improvements, measurable cost savings, and generally favorable incremental cost-effectiveness outcomes. Key determinants influencing economic viability included diagnostic performance, technology and implementation costs, workflow integration, and regional healthcare financing structures. Despite overall positive economic outcomes, methodological gaps remain, including incomplete cost evaluations, static modeling approaches, and limited consideration of equity. However, it is critical to differentiate clearly between findings derived from full economic evaluations and those from budget impact analyses, as these methods offer distinct yet complementary information. Full economic evaluations provide evidence regarding value for money and optimal resource allocation by simultaneously assessing incremental costs alongside clinical outcomes or quality-of-life measures, typically utilizing longer time horizons and broad societal perspectives. Conversely, budget impact analyses exclusively assess short-term affordability and immediate financial consequences for healthcare payers, facilitating near-term budgetary decision-making but without consideration of clinical effectiveness or broader societal benefits.

A central insight from our review is that AI interventions can generate economic benefits primarily by enhancing clinical performance. Many studies reported improvements in diagnostic accuracy and reductions in unnecessary procedures that translate directly into cost savings. For instance, evaluations in diabetic retinopathy screening and atrial fibrillation detection have shown that even modest improvements in sensitivity and specificity can result in lower per-patient screening costs and improved QALYs. This finding is consistent with Khanna et al., which underscores AI’s dual role in healthcare—enhancing clinical outcomes through improved diagnostic accuracy and personalized treatment while simultaneously reducing costs by optimizing resource allocation and streamlining care pathways^7^. Nonetheless, while these studies underscore AI’s potential, they often focus solely on operational savings, leaving out important elements like upfront capital expenditures, integration costs, and ongoing maintenance expenses. Without a complete net present value (NPV) analysis, estimates of economic benefit may be overly optimistic. It is important to note that the ICERs reported in many studies do not imply uniform economic benefits across all clinical applications. Instead, these findings underscore that the cost-effectiveness of AI is contingent upon specific contextual factors, including local healthcare settings, implementation costs, and diagnostic performance metrics.

Our review shows that nearly 79% of studies relied on decision-analytic models—primarily Markov models and decision trees—to simulate long-term outcomes. While these models provide a structured approach for economic evaluations, most of them used fixed transition probabilities that do not reflect the evolving performance of AI systems. Given that AI is designed to improve with exposure to larger datasets, there is a clear need for dynamic modeling approaches that can capture the adaptive learning curve inherent to these applications. Recent external literature emphasizes that models incorporating time-dependent parameters and learning curves provide a more accurate projection of long-term cost-effectiveness^39^.

A recurring limitation identified in our analysis is the incomplete accounting of costs. Many studies reported favorable ICERs or net savings primarily based on reduced diagnostic errors and streamlined workflows. However, these analyses often omitted substantial expenses such as initial investments in AI infrastructure, costs for software integration, and ongoing maintenance fees. Economic evaluations in fields like remote patient monitoring (RPM) and precision medicine often consider both upfront and recurring costs. While some full economic evaluations incorporate long-term cost-effectiveness analyses, including cost-utility and cost-benefit models, not all studies explicitly apply full net present value (NPV) calculations. Short-term studies may underestimate long-term financial sustainability, highlighting the need for broader economic perspectives and extended time horizons in future research^40^. A comprehensive cost evaluation is essential for decision-makers to assess whether the long-term savings from AI justify the initial expenditures. Incorporating all cost components into economic models would provide a more realistic picture of AI’s value proposition, enabling healthcare systems to allocate resources more judiciously.

In addition, while our analysis has focused on the direct costs associated with implementing clinical AI interventions, it is important to recognize that these estimates may underestimate the true cost burden of AI technologies. Recent evidence suggests that AI, particularly generative models, can be extraordinarily energy- and water-intensive, thereby incurring significant environmental costs^41^. This environmental impact not only contributes to the overall cost but may also have broader implications for sustainability and resource allocation in healthcare. Moreover, although several studies omit indirect costs and upfront investments—such as infrastructure and integration expenses—they simultaneously present many AI interventions as ‘dominant’ or cost-saving. This discrepancy suggests that the reported economic benefits may be overestimated.

Nearly all studies in our review incorporated sensitivity analyses, yet most focused only on parameter uncertainty and neglected structural uncertainty. While the majority of included studies conducted extensive sensitivity analyses to address parameter uncertainty, a significant limitation remains in the underexploration of structural uncertainty. Many evaluations rely on static models with fixed transition probabilities, which do not capture the dynamic learning and improvement of AI systems over time. This methodological shortcoming may result in overconfident estimates of cost savings, as the models do not account for potential shifts in clinical performance or unexpected cost escalations. Consequently, decision-makers should interpret these findings with caution, recognizing that the apparent economic benefits may be sensitive to untested structural assumptions. A recent review advocates for the use of advanced uncertainty analysis methods—such as Monte Carlo simulations and scenario analyses—that address both parameter and structural uncertainties^42^. By testing a range of assumptions and scenarios, these approaches provide decision-makers with a spectrum of potential outcomes and help identify the conditions under which AI interventions remain cost-effective. Enhancing uncertainty analysis will not only improve the credibility of individual studies but also strengthen the overall evidence base needed to guide investment in AI technologies.

One of the most concerning gaps in literature is the insufficient consideration of equity in economic evaluations. A notable shortcoming in the current literature is the limited disaggregation of economic outcomes by key demographic variables. This oversight raises concerns that the aggregate favorable findings may mask significant disparities in benefit distribution across different populations. Without subgroup-specific analyses, the potential for algorithmic bias remains unaddressed, and the risk is that cost-effectiveness results could disproportionately favor well-resourced groups. AI systems are highly dependent on the quality and representativeness of the data used for training. AI algorithms trained on non-representative datasets can amplify biases, leading to disparities in care and unequal model performance across populations. Models optimized for majority groups often perform poorly for underrepresented patients, increasing misdiagnoses and inequitable treatment recommendations^43,44^. Future evaluations should integrate equity metrics—such as subgroup-specific QALY gains and cost savings—to ensure that AI interventions deliver benefits equitably and that potential biases are identified and mitigated.

The economic evaluations in our review generally demonstrated high methodological rigor as evidenced by CHEERS and Drummond checklist scores, with clear study objectives, robust sensitivity analyses, and well-defined cost components. However, several limitations persist, including inconsistent reporting of indirect costs. It is important to note that many of the included studies omit indirect cost components and rely on static decision-analytic models. Such methodological choices may lead to an overestimation of the economic benefits of clinical AI interventions, as static models do not capture the dynamic improvements—such as learning curves and time-dependent performance enhancements—inherent to AI systems over time. The studies reviewed exhibit considerable heterogeneity in terms of methodologies, designs, and settings. This heterogeneity may limit the generalizability of our overall findings. Differences in study designs, population characteristics, and analytic methods necessitate a cautious interpretation of aggregated outcomes, as such variability can influence reported cost-effectiveness estimates. While the majority of studies report favorable economic outcomes for clinical AI interventions, the reliance on optimistic assumptions and methodological limitations in several studies suggests that these benefits may be overestimated. It is crucial that our conclusions adopt a more cautious tone, explicitly acknowledging that the true economic value of AI interventions remains uncertain until future evaluations address these limitations. These gaps underscore the need for future evaluations to adopt more comprehensive and adaptive methodologies to capture the evolving performance of AI systems and enhance the transparency and generalizability of findings across diverse healthcare settings.

Our study builds upon and extends the work of Voets et al.^18^ and Vithlani et al.^19^ by broadening the scope to encompass clinical AI interventions across multiple healthcare settings and specialties. While Voets et al. primarily examined automated imaging applications—highlighting limitations inherent to cost-minimization approaches—and Vithlani et al. emphasized the drawbacks of static decision-analytic models and incomplete reporting, our review confirms the generally favorable economic outcomes associated with AI while also revealing critical gaps. Notably, our findings underscore the need for dynamic modeling frameworks that capture the evolving performance of AI systems, comprehensive cost evaluations that account for implementation, integration, and maintenance expenditures, and robust equity analyses to ensure that economic benefits are equitably distributed. This integrated perspective not only reinforces the promise of AI in enhancing clinical performance and reducing costs but also provides a more nuanced understanding of its long-term economic viability.

The review’s strengths lie in its clear scope, rigorous adherence to PRISMA 2020 guidelines, and comprehensive search strategy across multiple databases, coupled with dual quality assessments using the CHEERS 2022 and Drummond checklists, which enhance its transparency and credibility. However, its weaknesses include variability in the methodologies and reporting standards of the included studies, a potential language bias from excluding non-English publications, and study heterogeneity. Although we used the Consolidated Health Economic Evaluation Reporting Standards (CHEERS 2022) to assess the quality of reporting across included studies, we recognize the subsequent release of the CHEERS-AI checklist^45^ in the third quarter of 2024 as a significant advancement. CHEERS-AI introduces 10 AI-specific reporting items and 8 elaborations on existing CHEERS 2022 domains, specifically addressing critical features such as AI learning over time, algorithmic bias, user autonomy, implementation requirements, and uncertainty arising from AI-specific factors. We believe its adoption in future reviews will meaningfully enhance the evaluation of AI-specific nuances in cost-effectiveness research. The emergence of CHEERS-AI also highlights the need for ongoing refinement of reporting standards to remain responsive to rapidly evolving digital health technologies. Although we assessed quality using the Drummond checklist, we acknowledge that it is somewhat dated and has been critiqued in recent literature. Newer alternatives, such as the CHEQUE checklist^46^, have been proposed; however, no single quality checklist for cost-effectiveness analyses is universally accepted.

Future research in the economic evaluation of clinical AI interventions should prioritize several key areas. Dynamic adaptive modeling techniques are essential for accurately capturing AI’s evolving clinical and economic performance over time. However, implementing these approaches faces obstacles such as limited availability of longitudinal data, computational complexity, and resource constraints, particularly in lower-resource settings^47^. These barriers can be overcome by promoting international collaborative research networks, investing in robust data-sharing infrastructures, and providing specialized methodological training to build analytical capacity globally.

A further priority is adopting comprehensive standardized cost evaluation frameworks. Such frameworks should include thorough NPV analyses, clearly accounting for initial investments, integration costs, and ongoing maintenance expenses. Potential challenges here include inconsistent definitions and cost-reporting practices, variability in regional standards, and resistance to standardization^48^. Strategies to mitigate these obstacles include encouraging global adherence to standards such as CHEERS 2022^49^ and CHEERS-AI^45^, facilitating international consensus-building forums, and standardizing reporting practices to enhance transparency and comparability across healthcare systems.

Researchers should also adopt advanced uncertainty analysis techniques, such as Monte Carlo simulations and comprehensive scenario analyses, addressing both parameter and structural uncertainties. Implementation barriers, such as methodological complexity and limited technical expertise, especially in resource-limited contexts, can be overcome by developing international methodological training programs, sharing analytical resources and software through global collaborations, and providing detailed methodological guidelines to support consistent application worldwide.

Future research should integrate a distributional cost-effectiveness analysis framework to explicitly assess how the costs and benefits of clinical AI interventions are allocated across different population subgroups. This approach enables decision-makers to weigh tradeoffs between efficiency and equity, ensuring that AI-driven innovations deliver benefits that are shared fairly^50,51^.

It is also critical to embed equity considerations throughout the AI development process—from initial model design and data curation to clinical integration and post-deployment monitoring. By proactively integrating equity metrics at each phase, researchers can identify and mitigate biases, thereby promoting equitable outcomes across diverse demographic groups^52^.

Moreover, future evaluations must incorporate assessments of algorithmic bias and explicitly examine AI’s impact on various demographic populations^53,54^. Addressing challenges such as the scarcity of representative datasets and the lack of standardized equity frameworks will require establishing international standards for inclusive data collection, mandating equity audits and bias-aware frameworks, and promoting policy-driven initiatives to encourage equitable implementation globally.

Finally, conducting context-specific economic evaluations tailored to local healthcare infrastructures, reimbursement mechanisms, and regional cost variations is crucial. Obstacles such as variations in regional healthcare financing, infrastructural disparities, and lack of suitable evaluation frameworks must be tackled through strategies like creating adaptable economic evaluation models, facilitating regional benchmarking studies, and developing localized policy guidance to support context-specific decision-making.

Dynamic and adaptive modeling, coupled with comprehensive cost analyses, will yield more accurate and realistic long-term projections that truly reflect the evolving nature of AI technologies. By integrating clinical and economic outcomes and prioritizing equity, future studies will ensure that AI advancements not only enhance patient care but also deliver sustainable and fair economic benefits.

Tailored, context-specific evaluations will empower healthcare policymakers to make informed, regionally relevant decisions, fostering efficient resource allocation and smoother technology adoption. Moreover, standardized methodologies and interdisciplinary collaborations will strengthen the evidence base, ensuring that the economic evaluations of clinical AI are both robust and actionable. Ultimately, these strategies will drive investments and policy decisions that not only elevate the quality of patient care but also secure long-term economic sustainability and equitable access across global healthcare systems.

The feasibility and implications of these strategies vary substantially across healthcare systems and geographical regions. In high-resource settings where advanced healthcare infrastructures, sophisticated technological capabilities, and established regulatory frameworks exist, obstacles to implementation primarily include complex integration processes, high initial AI technology costs, and regulatory approval complexities. These regions can overcome these challenges by leveraging public-private partnerships, enhancing regulatory clarity and guidelines, and developing clear reimbursement policies incentivizing quality-driven AI adoption.

Conversely, in low-or -middle resource settings significant barriers include insufficient data infrastructures, limited financial resources, and scarce local technical expertise. These challenges can be addressed through targeted initiatives, including international support for capacity-building, investment in scalable, low-cost AI technologies, establishment of global resource-sharing networks, and development of tailored evaluation frameworks sensitive to local economic contexts.

Therefore, tailored strategies and international collaboration remain crucial to overcoming region-specific barriers, ensuring clinical AI delivers substantial, equitable, and sustainable health-economic benefits across diverse global healthcare environments.

In conclusion, this review finds that clinical AI interventions are generally associated with improved diagnostic accuracy, measurable gains in quality-adjusted life years, and the potential for meaningful cost savings compared to conventional care. However, the strength of this evidence is constrained by notable methodological limitations across literature, most significantly, the frequent omission of essential cost components such as infrastructure investment, system integration, workforce training, and long-term maintenance. These omissions introduce uncertainty and suggest that the reported economic value may, in some cases, be overstated. To generate more accurate and policy-relevant insights, future evaluations must adopt dynamic, longitudinal, and fully transparent modeling frameworks that comprehensively account for both direct and indirect costs over time.

## Methods

This systematic review was conducted in accordance with the Preferred Reporting Items for Systematic Reviews and Meta-Analyses (PRISMA) 2020 statement^55^. To ensure the relevance and quality of the included studies, specific inclusion and exclusion criteria were established (Table 2).

**Table 2:** Inclusion and exclusion criteria

Criterion	Inclusion	Exclusion
Population	Studies in clinical settings where AI is implemented in patient care (e.g., hospital, primary care, community settings)	Non‑clinical settings; studies focusing solely on technical algorithm development without clinical application
Intervention	Use of AI technologies (machine learning, deep learning, or related methods) integrated into clinical decision making or workflow	Studies reporting only on algorithm performance without economic evaluation
Outcomes	Reporting of economic endpoints (cost‑effectiveness, cost‑utility, cost‑benefit, budget impact, etc.)	Studies without any economic outcome data
Study design	Peer reviewed, primary research: RCTs, observational studies, decision analytic models, qualitative or mixed‑methods studies	Reviews, editorials, commentaries, and abstracts without full-text data
Language	English	Non‑English publications

### Search strategy

A comprehensive literature search was conducted across major electronic databases: PubMed/MEDLINE, Embase, Web of Science, and the Cochrane Library. The search was designed to capture all relevant studies published from database inception to January 2025. We developed a detailed search strategy that combined both controlled vocabulary (e.g., MeSH, Emtree) and free-text terms. The core concepts in the search strategy included: (1) artificial intelligence and its synonyms (“artificial intelligence”, “machine learning”, “deep learning”, “AI”), (2) clinical application domains (e.g., “diagnostic”, “prognostic”, “treatment planning”, “workflow automation”, “clinical decision support”), and (3) economic evaluation terminology (e.g., “cost-effectiveness”, “cost-utility”, “cost-benefit”, “budget impact”, “economic evaluation”). The search strings were tailored to the syntax and subject headings of each database (Table 3).

**Table 3:** Search strategy

Database	Search string
PubMed/MEDLINE	(“Artificial Intelligence”[MeSH] OR “machine learning” OR “deep learning” OR “AI”) AND (“Cost Effectiveness”[MeSH] OR “cost-effectiveness” OR “cost utility” OR “budget impact” OR “economic evaluation”) AND (clinical OR healthcare OR “patient care”)
Embase	(‘artificial intelligence’/exp OR “machine learning” OR “deep learning” OR “AI”) AND (“cost effectiveness” OR “cost utility” OR “budget impact” OR “economic evaluation”) AND (clinical OR healthcare)
Web of Science	TS = (“artificial intelligence” OR “machine learning” OR “deep learning” OR “AI”) AND TS = (“cost-effectiveness” OR “cost utility” OR “budget impact” OR “economic evaluation”) AND TS = (clinical OR healthcare OR “patient care”)
Cochrane Library	(“Artificial Intelligence” OR “machine learning” OR “deep learning” OR “AI”) in Title, Abstract, Keywords AND (“cost-effectiveness” OR “cost utility” OR “budget impact” OR “economic evaluation”) in Title, Abstract, Keywords

### Study selection

The records identified through comprehensive database searches were imported into Rayyan, a specialized systematic review screening platform^56^. Two reviewers independently screened titles and abstracts against the predetermined eligibility criteria, and full texts of potentially eligible studies were subsequently retrieved and assessed independently. Any discrepancies in the screening process were resolved by consensus or, if necessary, by consultation with a third reviewer. A summary of records identified, screened, excluded (with reasons), and included is provided in the Results section.

### Data extraction

Data extraction was carried out using a standardized form developed in Microsoft Excel and piloted on a subset of studies to ensure consistency and comprehensiveness. From each study, we extracted detailed information including study identification (author(s), publication year, and country), clinical domain and setting (such as hospital, primary care, or community clinics, with examples including acute stroke, oncology, and diabetic retinopathy), and study design (e.g., randomized controlled trials, observational studies, or decision analytic models). Additionally, we recorded detailed descriptions of the AI intervention and the comparator (standard care or an alternative technology), as well as specifics of the economic evaluation, including the type of analysis (cost-effectiveness, cost-utility, cost-benefit, or budget impact), the perspective adopted (healthcare system, societal, or payer), the time horizon, discounting methods, and the key outcome measures such as incremental cost-effectiveness ratios, quality-adjusted life years, and cost savings. Indirect cost valuation specifically refers to estimating costs associated with productivity losses due to illness or healthcare interventions, such as lost earnings from work absences or reduced productivity, typically assessed using methods like the human capital or friction cost approach^57,58^. Principal economic findings and study limitations were also meticulously documented. To enhance comparability and transparency, all economic evaluations included in this review have been standardized by explicitly specifying the methods used for currency conversion and the cost-year baselines. Where such details were not provided in the original studies, we have applied standard conversion factors and assumed a baseline consistent with international guidelines. Data extraction was independently performed by two reviewers, and any discrepancies were resolved through discussion to ensure accuracy and reliability.

### Data analysis

Data analysis was conducted through a thematic synthesis approach, following the methodologies outlined by Braun and Clarke^59^ and Thomas and Harden^60^. Initially, all extracted data regarding economic outcomes and study characteristics were coded using descriptive labels, which included recurring themes such as cost savings, incremental cost-effectiveness ratios (ICER) thresholds, diagnostic accuracy, and model assumptions. These codes were then aggregated into broader descriptive themes that captured key dimensions of economic performance across the studies, including “Cost Drivers,” “Outcome Measures,” and “Contextual Influences.” Building upon these descriptive themes, analytical themes were developed to interpret the overarching economic impact of clinical AI interventions. For example, initial codes such as ‘reduction in unnecessary procedures,’ ‘cost savings per patient,’ and ‘improved diagnostic accuracy leading to cost-effectiveness’ were grouped under the descriptive theme of “Comparative Economic Outcomes.” Further, analytical synthesis identified deeper insights such as the context-dependent nature of AI economic benefits, subsequently forming the higher-order analytical theme “Economic Impact and Implementation Considerations. The thematic synthesis process was facilitated by NVivo software (version 14, QSR International)^61^, which provided a systematic platform for coding, organizing, and visualizing themes. Two reviewers independently conducted the thematic synthesis to ensure robustness, and any discrepancies in theme development were resolved through discussion. We also examined regional context as a proxy for structural equity by extracting geographic, infrastructural, and healthcare system characteristics that may influence equitable access to AI technologies. Particular attention was given to studies conducted in low-resource, rural, or underserved settings, where disparities in digital infrastructure or workforce capacity could either exacerbate or reduce healthcare inequities. These contextual attributes were analyzed alongside explicitly reported equity-related indicators—such as demographic subgroup analyses or targeted interventions for vulnerable populations—to explore how economic value and access to AI interventions may vary across settings.

This review refers to clinical AI interventions to artificial intelligence technologies that are implemented to support, augment, or replace specific aspects of clinical decision-making in healthcare settings. This definition explicitly includes hybrid interventions, wherein AI systems operate in conjunction with human oversight as well as standalone AI applications. The synthesis process enabled the identification of patterns across various clinical domains, highlighting both the generalizable economic benefits—such as consistent cost savings observed in screening strategies—and the context-dependent variability, such as differences in cost-effectiveness thresholds among different countries. Two reviewers independently conducted the thematic synthesis to ensure robustness, and any discrepancies in theme development were resolved through discussion. The final synthesis accurately reflects the economic evidence across the 19 included studies and is presented in the Results section.

To ensure a rigorous evaluation of the included economic evaluations, we independently appraised each study using two established instruments: the Consolidated Health Economic Evaluation Reporting Standards (CHEERS) 2022 checklist^49^ and the Drummond checklist^62^. The updated CHEERS 2022 checklist consists of 28 items that span key reporting domains, including title and abstract, background, objectives, target population, setting, study perspective, comparators, time horizon, discounting, outcome measures, resource use, cost estimation, model assumptions, analytic methods (including sensitivity analyses), and funding and conflict of interest disclosures. Two independent reviewers evaluated each study against these 28 items, scoring each item as “adequately reported” (1) or “not adequately reported” (0). The total score for each study was then calculated and expressed as “X/28.” Discrepancies between reviewers were resolved by consensus, and a third reviewer was consulted if needed. A detailed item breakdown is provided in Supplementary Table 2. The Drummond checklist focuses on methodological quality and risk of bias of economic evaluations. It consists of 10 key items, which include:

1. Whether the research question was clearly stated,
2. Whether the alternatives (including the AI intervention and comparators) were clearly described,
3. Whether clinical effectiveness was established,
4. Whether all relevant costs and outcomes were identified,
5. Whether costs and outcomes were measured accurately,
6. Whether costs and outcomes were valued credibly,
7. Whether an incremental analysis was performed,
8. Whether uncertainty was adequately addressed,
9. Whether heterogeneity was explored,
10. Whether the conclusions were justified by the evidence.

Similar to the CHEERS assessment, each item on the Drummond checklist was rated as “fully met” (1) or “not fully met” (0). The scores for each study were summed to provide a total score out of 10 (e.g., “9/10” indicates that 9 of the 10 items were fully met). Using these two checklists allows us to comprehensively assess both the reporting quality (via CHEERS) and the methodological rigor/risk of bias (via Drummond) of each included study. These scores were then used to inform our synthesis and discussion of the overall quality of the economic evidence. Discrepancies between reviewers were resolved by consensus, and a third reviewer was consulted if needed. The scoring outcomes were explicitly considered when interpreting findings. Studies achieving higher CHEERS and Drummond scores were regarded as more reliable, methodologically rigorous, and transparent in reporting. Conversely, lower-scoring studies indicated potential limitations, including inadequate reporting of critical cost components, incomplete justification of model assumptions, or insufficient sensitivity analysis. Such limitations necessitated cautious interpretation of their results within the thematic synthesis. To illustrate, a study scoring below 8/10 on the Drummond checklist or below 23/28 on CHEERS would prompt a critical appraisal of its contributions, particularly when integrating findings into broader conclusions or recommendations.

## Supplementary information

Supplemental information

## Data Availability

All data analyzed in this review were extracted from the studies cited in the References. A full extraction table is available in Supplementary Material.

## Abbreviations

AI: Artificial Intelligence
PRISMA: Preferred Reporting Items for Systematic Reviews and Meta-Analyses
CHEERS: Consolidated Health Economic Evaluation Reporting Standards
RCT: Randomized Controlled Trial
CEA: Cost-Effectiveness Analysis
CUA: Cost-Utility Analysis
CMA: Cost-Minimization Analysis
BIA: Budget Impact Analysis
MeSH: Medical Subject Headings
Emtree: (Embase’s controlled vocabulary)
ML: Machine Learning
AF: Atrial Fibrillation
QALY: Quality-Adjusted Life Year(s)
ICER: Incremental Cost-Effectiveness Ratio
ROI: Return on Investment
PMPM: Per Member Per Month
LDCT: Low-Dose Computed Tomography
CXR: Chest X-ray
ICU: Intensive Care Unit
NHS: National Health Service
DALY: Disability-Adjusted Life Year
NPV: Net Present Value
AUROC: Area Under the Receiver Operating Characteristic

## Code availability

The codes used in the analysis of this study will be made available from the corresponding author upon reasonable request.
