# Supplementary material for: Systematic review of cost effectiveness and budget impact of artificial intelligence in healthcare
Source: NPJ Digit Med. 2025 Aug 26;8:548. doi: 10.1038/s41746-025-01722-y (PMC12381244; doi:10.1038/s41746-025-01722-y)
Supplement: Supplementary file 1 — Supplemental information [file 41746_2025_1722_MOESM1_ESM.pdf]

Supplementary Table 1. Summary of Studies Categorized by Modeling Approach

Study (Author, Year)	Intervention/Focus Area	Model Type / Economic Evaluation Methodology	Static vs. Dynamic
Murtojärvi et al. (2020)	Metastatic castration-resistant prostate cancer	Prognostic models using Greedy and LASSO feature selection	Static
Hill et al. (2020)	Atrial fibrillation screening using an ML risk prediction algorithm	ML risk prediction algorithm using clinical and demographic data	Dynamic
Mori et al. (2020)	AI-aided polyp diagnosis in colonoscopy	Cost-minimization analysis based on clinical trial data	Static
Hendrix et al. (2022)	Economic evaluation framework for clinical AI intervention	Theoretical analysis and conceptual framework development that outlines multiple evaluation methods (CEA, CUA, CBA, CMA) as applied to AI	Static
Karabeg et al. (2024)	AI (EyeArt®) vs. ophthalmologist for DR screening in minority women	Cost-minimization analysis for a single screening episode	Static
Huang et al. (2022)	AI screening for diabetic retinopathy in rural China	Hybrid decision tree and Markov model	Dynamic
Schwendicke et al. (2021)	AI for proximal caries detection	Markov model with Monte Carlo microsimulations	Dynamic
Areia et al. (2022)	AI-assisted colonoscopy screening for colorectal cancer	Markov model microsimulation study	Dynamic
de Vos et al. (2022)	AI tool to prevent untimely ICU discharge	Cost-utility analysis using a 7-state Markov model	Dynamic
Ericson et al. (2022)	Early detection of sepsis in ICUs	Decision tree–based health economic modeling	Static
Kessler et al. (2021)	AI-assisted medication management in a Medicaid population	Retrospective observational analysis using regression methods	Static
Mital & Nguyen (2022)	AI vs. polygenic risk score for breast cancer screening	Hybrid decision tree/microsimulation model	Dynamic
Nsengiyumva et al. (2021)	AI-based chest X-ray triage for TB symptoms	Decision analysis model (decision tree)	Static
Salcedo et al. (2021)	AI monitoring for active tuberculosis treatment adherence	Markov model-based cost-effectiveness analysis	Dynamic

Study (Author, Year)	Intervention/Focus Area	Model Type / Economic Evaluation Methodology	Static vs. Dynamic
van Leeuwen et al. (2021)	AI-aided vessel occlusion detection in acute stroke	Markov model-based cost-effectiveness analysis	Dynamic
Xiao et al. (2021)	AI-assisted glaucoma screening in rural China	Markov model–based health economic analysis	Dynamic
Ziegelmayer et al. (2022)	AI-assisted lung cancer screening with LDCT	Markov model–based cost-effectiveness analysis with probabilistic sensitivity analysis	Dynamic
Gomez Rossi et al. (2022)	AI as decision-support for melanoma, dental caries, & diabetic retinopathy	Markov model–based cost-effectiveness analysis (lifetime modeling)	Dynamic
Szymanski et al. (2022)	ML algorithm for AF risk prediction (budget impact analysis)	National population model over a 3-year projection	Dynamic

Abbreviation Legend:

- **AI** – Artificial Intelligence
- **ML** – Machine Learning
- **DR** – Diabetic Retinopathy
- **ICU** – Intensive Care Unit
- **LDCT** – Low-Dose Computed Tomography
- **TB** – Tuberculosis
- **CEA** – Cost-Effectiveness Analysis
- **CUA** – Cost-Utility Analysis
- **CBA** – Cost-Benefit Analysis
- **CMA** – Cost-Minimization Analysis
- **Static Models** – Economic evaluation models with fixed parameters (i.e., no adaptive learning or time-dependent changes)
- **Dynamic Models** – Economic evaluation models incorporating adaptive features, such as learning curves or time-dependent transition probabilities

Supplementary Table 2: CHEERS 2022 Checklist for the 19 Included Studies

Study	CHEERS Items Adequately Reported (out of 28)	Key Reporting Strengths	Key Reporting Limitations
Areia et al., 2022	25/28	Clear objectives; comprehensive methods; detailed sensitivity analyses and outcome reporting	Minor details on currency conversion and cost year not fully described
de Vos et al., 2022	24/28	Well-defined perspective; detailed cost components; clear comparator description	Limited reporting on indirect costs in some sections
Ericson et al., 2022	23/28	Robust description of effectiveness data; clear time horizon; adequate sensitivity analyses	Limited details on utility measurement and valuation
Gomez Rossi et al., 2022	27/28	Detailed reporting across specialties; clear incremental analysis and outcomes	Slight variability in reporting subgroup heterogeneity
Hendrix et al., 2022	24/28	Comprehensive framework; clear assumptions; transparent multi-perspective reporting	Conceptual approach with less granular numerical detail
Hill et al., 2020	25/28	Clear comparator descriptions; robust sensitivity analyses; detailed screening strategy	Minor omissions in discounting method details
Huang et al., 2022	23/28	Thorough description of methods and outcomes; clear perspective and time horizon	Some uncertainty regarding currency conversion details and cost year
Karabeg et al., 2024	21/28	Clear description of setting and population; direct comparison of AI vs. human grading	Small sample size limits detailed heterogeneity reporting
Kessler et al., 2021	24/28	Detailed cost estimation; clear perspective; comprehensive methods	Limited discussion regarding underlying model assumptions

Mital & Nguyen, 2022	25/28	Comprehensive reporting of screening strategy and incremental results; clear comparator definition	Some variability in reporting valuation of preference-based outcomes
Mori et al., 2020	24/28	Detailed methodology; clear cost components; well-reported sensitivity analyses	Certain aspects of the sensitivity analyses could be more elaborated
Murtojärvi et al., 2020	24/28	Clear definition of target population; explicit reporting of feature selection methods	Some missing details on indirect costs and model assumptions
Nsengiyumva et al., 2021	23/28	Clear reporting of comparators and cost estimation; adequate analytic approach	Limited subgroup analyses; fewer details on indirect cost components
Salcedo et al., 2021	23/28	Adequate description of effectiveness and cost components; clear incremental analysis	Short time horizon limits reporting of long-term outcomes
Schwendicke et al., 2021	25/28	Comprehensive outcome measurement; detailed discounting; robust sensitivity analyses	Minor omissions regarding indirect cost reporting
Szymanski et al., 2022	24/28	Good reporting of model inputs; extensive sensitivity analyses; clear alternatives	Some aspects of heterogeneity not deeply discussed
van Leeuwen et al., 2021	24/28	Detailed reporting of cost, effectiveness, and discounting parameters; clear incremental analysis	Limited validation of long-term projections; some assumptions need further detail
Xiao et al., 2021	23/28	Clear description of screening strategy and outcomes; adequate cost reporting	Some uncertainty in reporting capital cost details and conversion methods
Ziegelmayer et al., 2022	25/28	Extensive sensitivity and threshold analyses; clear reporting of cost and	Some variability in reporting specific model assumptions,

		outcome measures with robust modelling	requiring further empirical validation

Supplementary Table 3: Drummond Checklist for the 19 Included Studies

Study	Drummond Items Fully Met (out of 10)	Key Methodological Strengths	Key Methodological Limitations
Areia et al., 2022	9/10	Clearly defined alternatives; robust incremental analysis; thorough sensitivity testing	Some uncertainty in cost valuation methods
de Vos et al., 2022	8/10	Well-described comparators; established effectiveness data	Certain cost assumptions not fully justified
Ericson et al., 2022	9/10	Detailed incremental analysis; robust sensitivity analyses; appropriate outcome measurement	Limited valuation of indirect costs
Gomez Rossi et al., 2022	9/10	Clear identification of costs and outcomes; detailed incremental analyses across specialties	Variability in outcome measurement for diabetic retinopathy
Hendrix et al., 2022	8/10	Comprehensive identification of alternatives; clear listing of assumptions	Conceptual framework lacks granular quantitative valuation
Hill et al., 2020	9/10	Effective measurement and incremental analysis; strong sensitivity testing	Minor limitations in valuation of certain cost components
Huang et al., 2022	8/10	Well-established effectiveness data; robust modelling	Some details of cost and outcome valuation are less clearly described
Karabeg et al., 2024	8/10	Clear description of alternatives; effective incremental analysis	Limited sample size reduces robustness and generalizability
Kessler et al., 2021	9/10	Comprehensive cost and outcome measurement; robust regression analysis	Retrospective design may introduce bias
Mital & Nguyen, 2022	9/10	Detailed incremental analysis; robust sensitivity testing; clear comparator definition	Model assumptions are specific to a single population
Mori et al., 2020	8/10	Innovative prognostic modelling with effective incremental analysis	Some measurement details (e.g., missing variables) are less elaborated
Murtojärvi et al., 2020	8/10	Clear definition of target population; explicit reporting of feature selection methods; effective incremental analysis	Some missing details on indirect cost components and full justification of model assumptions
Nsengiyumva et al., 2021	8/10	Clear establishment of effectiveness and cost measures; robust sensitivity analysis	Short time horizon and omission of some indirect cost components

Salcedo et al., 2021	8/10	Clear incremental analysis and outcome measurement; comprehensive sensitivity testing	Limited follow-up reduces assessment of long-term benefits
Schwendicke et al., 2021	9/10	Thorough identification and measurement of costs and outcomes; strong incremental analysis	Some limitations in reporting indirect costs
Szymanski et al., 2022	8/10	Robust incremental analysis; extensive sensitivity testing; clear description of alternatives	Reliance on assumptions regarding screening uptake and participation rates
van Leeuwen et al., 2021	9/10	Excellent incremental analysis and robust uncertainty assessment; clear outcome measurement	Some long-term projection assumptions require further validation
Xiao et al., 2021	8/10	Clear identification of cost and outcome components; effective incremental analysis	Some limitations in detailed measurement of capital costs
Ziegelmayer et al., 2022	9/10	Robust incremental and sensitivity analyses; comprehensive cost and outcome measurement	Certain model assumptions require additional empirical validation

PRISMA 2020 Checklist

Section and Topic	Item #	Checklist item	Location where item is reported
**TITLE**			
Title	1	Identify the report as a systematic review.	1
**ABSTRACT**			
Abstract	2	See the PRISMA 2020 for Abstracts checklist.	2
**INTRODUCTION**			
Rationale	3	Describe the rationale for the review in the context of existing knowledge.	3,4,5
Objectives	4	Provide an explicit statement of the objective(s) or question(s) the review addresses.	5
**METHODS**			
Eligibility criteria	5	Specify the inclusion and exclusion criteria for the review and how studies were grouped for the syntheses.	25
Information sources	6	Specify all databases, registers, websites, organisations, reference lists and other sources searched or consulted to identify studies. Specify the date when each source was last searched or consulted.	25,26
Search strategy	7	Present the full search strategies for all databases, registers and websites, including any filters and limits used.	26
Selection process	8	Specify the methods used to decide whether a study met the inclusion criteria of the review, including how many reviewers screened each record and each report retrieved, whether they worked independently, and if applicable, details of automation tools used in the process.	26,27
Data collection process	9	Specify the methods used to collect data from reports, including how many reviewers collected data from each report, whether they worked independently, any processes for obtaining or confirming data from study investigators, and if applicable, details of automation tools used in the process.	26.27.28
Data items	10a	List and define all outcomes for which data were sought. Specify whether all results that were compatible with each outcome domain in each study were sought (e.g. for all measures, time points, analyses), and if not, the methods used to decide which results to collect.	24
	10b	List and define all other variables for which data were sought (e.g. participant and intervention characteristics, funding sources). Describe any assumptions made about any missing or unclear information.	25
Study risk of bias assessment	11	Specify the methods used to assess risk of bias in the included studies, including details of the tool(s) used, how many reviewers assessed each study and whether they worked independently, and if applicable, details of automation tools used in the process.	29,30
Effect measures	12	Specify for each outcome the effect measure(s) (e.g. risk ratio, mean difference) used in the synthesis or presentation of results.	26
Synthesis methods	13a	Describe the processes used to decide which studies were eligible for each synthesis (e.g. tabulating the study intervention characteristics and comparing against the planned groups for each synthesis (item #5)).	26,27
	13b	Describe any methods required to prepare the data for presentation or synthesis, such as handling of missing summary statistics, or data conversions.	27
	13c	Describe any methods used to tabulate or visually display results of individual studies and syntheses.	25
	13d	Describe any methods used to synthesize results and provide a rationale for the choice(s). If meta-analysis was performed, describe the model(s), method(s) to identify the presence and extent of statistical heterogeneity, and software package(s) used.	27,28
	13e	Describe any methods used to explore possible causes of heterogeneity among study results (e.g. subgroup analysis, meta-regression).	28
	13f	Describe any sensitivity analyses conducted to assess robustness of the synthesized results.	26
Reporting bias assessment	14	Describe any methods used to assess risk of bias due to missing results in a synthesis (arising from reporting biases).	27
Certainty assessment	15	Describe any methods used to assess certainty (or confidence) in the body of evidence for an outcome.	27

PRISMA 2020 Checklist

Section and Topic	Item #	Checklist item	Location where item is reported
**RESULTS**			
Study selection	16a	Describe the results of the search and selection process, from the number of records identified in the search to the number of studies included in the review, ideally using a flow diagram.	5,6
	16b	Cite studies that might appear to meet the inclusion criteria, but which were excluded, and explain why they were excluded.	5
Study characteristics	17	Cite each included study and present its characteristics.	5,6
Risk of bias in studies	18	Present assessments of risk of bias for each included study.	16,17
Results of individual studies	19	For all outcomes, present, for each study: (a) summary statistics for each group (where appropriate) and (b) an effect estimate and its precision (e.g. confidence/credible interval), ideally using structured tables or plots.	6-11
Results of syntheses	20a	For each synthesis, briefly summarise the characteristics and risk of bias among contributing studies.	12
	20b	Present results of all statistical syntheses conducted. If meta-analysis was done, present for each the summary estimate and its precision (e.g. confidence/credible interval) and measures of statistical heterogeneity. If comparing groups, describe the direction of the effect.	NA
	20c	Present results of all investigations of possible causes of heterogeneity among study results.	12,13
	20d	Present results of all sensitivity analyses conducted to assess the robustness of the synthesized results.	13,14
Reporting biases	21	Present assessments of risk of bias due to missing results (arising from reporting biases) for each synthesis assessed.	15,16,17
Certainty of evidence	22	Present assessments of certainty (or confidence) in the body of evidence for each outcome assessed.	16,17
**DISCUSSION**			
Discussion	23a	Provide a general interpretation of the results in the context of other evidence.	17,18
	23b	Discuss any limitations of the evidence included in the review.	20,21
	23c	Discuss any limitations of the review processes used.	18,19
	23d	Discuss implications of the results for practice, policy, and future research.	22,23,24
**OTHER INFORMATION**			
Registration and protocol	24a	Provide registration information for the review, including register name and registration number, or state that the review was not registered.	review was not registered
	24b	Indicate where the review protocol can be accessed, or state that a protocol was not prepared.	NA
	24c	Describe and explain any amendments to information provided at registration or in the protocol.	NA
Support	25	Describe sources of financial or non-financial support for the review, and the role of the funders or sponsors in the review.	32
Competing interests	26	Declare any competing interests of review authors.	32
Availability of data, code and other materials	27	Report which of the following are publicly available and where they can be found: template data collection forms; data extracted from included studies; data used for all analyses; analytic code; any other materials used in the review.	31

PRISMA 2020 Checklist

From: Page MJ, McKenzie JE, Bossuyt PM, Boutron I, Hoffmann TC, Mulrow CD, et al. The PRISMA 2020 statement: an updated guideline for reporting systematic reviews. BMJ 2021;372:n71. doi: 10.1136/bmj.n71. This work is licensed under CC BY 4.0. To view a copy of this license, visit https://creativecommons.org/licenses/by/4.0/
